# Using an intervention mapping approach for planning, implementing and assessing a community-led project towards malaria elimination in the Eastern Province of Rwanda

**DOI:** 10.1186/s12936-016-1645-3

**Published:** 2016-12-16

**Authors:** Chantal Marie Ingabire, Emmanuel Hakizimana, Fredrick Kateera, Alexis Rulisa, Bart Van Den Borne, Ingmar Nieuwold, Claude Muvunyi, Constantianus J. M. Koenraadt, Michele Van Vugt, Leon Mutesa, Jane Alaii

**Affiliations:** 1 Medical Research Center, Rwanda Biomedical Center, Kigali, Rwanda; 2 Department of Health Promotion, Maastricht University, Maastricht, The Netherlands; 3 Malaria and Other Parasitic Diseases Division, Rwanda Biomedical Center, Kigali, Rwanda; 4 Wageningen University, Wageningen, The Netherlands; 5 Academic Medical Center, Amsterdam, The Netherlands; 6 Department of Cultural Anthropology and Development Studies, Radboud University Nijmegen, Nijmegen, The Netherlands; 7 Foundation the 100th Village, Amsterdam, The Netherlands; 8 College of Medicine and Health Sciences, University of Rwanda, Butare, Rwanda; 9 Context Factor Solutions, Nairobi, Kenya

**Keywords:** Community, Engagement, Involvement, Empowerment, Participation, Intervention mapping, Malaria, Rwanda

## Abstract

**Background:**

Active community participation in malaria control is key to achieving malaria pre-elimination in Rwanda. This paper describes development, implementation and evaluation of a community-based malaria elimination project in Ruhuha sector, Bugesera district, Eastern province of Rwanda.

**Methods:**

Guided by an intervention mapping approach, a needs assessment was conducted using household and entomological surveys and focus group interviews. Data related to behavioural, epidemiological, entomological and economical aspects were collected. Desired behavioural and environmental outcomes were identified concurrently with behavioural and environmental determinants. Theoretical methods and their practical applications were enumerated to guide programme development and implementation. An operational plan including the scope and sequence as well as programme materials was developed. Two project components were subsequently implemented following community trainings: (1) community malaria action teams (CMATs) were initiated in mid-2014 as platforms to deliver malaria preventive messages at village level, and (2) a mosquito larval source control programme using biological substances was deployed for a duration of 6 months, implemented from January to July 2015. Process and outcome evaluation has been conducted for both programme components to inform future scale up.

**Results:**

The project highlighted malaria patterns in the area and underpinned behavioural and environmental factors contributing to malaria transmission. Active involvement of the community in collaboration with CMATs contributed to health literacy, particularly increasing ability to make knowledgeable decisions in regards to malaria prevention and control. A follow up survey conducted six months following the establishment of CMATs reported a reduction of presumed malaria cases at the end of 2014. The changes were related to an increase in the acceptance and use of available preventive measures, such as indoor residual spraying and increase in community-based health insurance membership, also considered as a predictor of prompt and adequate care. The innovative larval source control intervention contributed to reduction in mosquito density and nuisance bites, increased knowledge and skills for malaria control as well as programme ownership.

**Conclusion:**

This community-based programme demonstrated the feasibility and effectiveness of active community participation in malaria control activities, which largely contributed to community empowerment and reduction of presumed malaria in the area. Further studies should explore how gains may be sustained to achieve the goal of malaria pre-elimination.

## Background

Malaria remains a major public health problem worldwide [1]. The World Health Organization (WHO) estimates about 3.2 million people to be at risk of malaria worldwide and in 2015, 89% of malaria cases and 91% of malaria deaths of the global malaria burden were located in sub Saharan Africa [1]. However, between 2000 and 2015 malaria incidence declined significantly by 37% globally, leading to the achievement of the sixth millennium development goal of halving and reversing malaria incidence by 2015 [1]. Malaria incidence in 57 countries have reduced by 75% as per the World Health Assembly’s target [1, 2]. In sub-Saharan Africa, malaria control interventions such as long lasting insecticide-treated nets (LLINs) and indoor residual spraying (IRS) accounted for 70% of the 943 million fewer malaria cases occurring between 2001 and 2015 thus preventing 663 million malaria cases [1].

In Rwanda, an 86% reduction in malaria incidence and a 74% reduction in malaria mortality were observed from 2005 to 2012 [3]. Despite these gains, malaria control is deemed fragile following upsurges in 2009, 2012, 2013 and recently in 2015. Nevertheless, the country is aiming at malaria pre-elimination status by 2018 thus shifting from malaria control to surveillance, investigation, and response [3]. To achieve this, the country has set various objectives including effective protection with vector control strategies, prompt testing and treatment, multi-sectoral partnership and promotion of correct knowledge and practices towards malaria preventive measures [3].

Malaria elimination will require targeting the parasite (plasmodium), the vector (mosquito) and most importantly the human host, hence community participation in malaria control is perceived as a key factor [4]. Community participation focuses on the involvement of a defined community in problem identification and the creation of conditions of change for both individuals and groups [5]. Thus, an effective implementation of community-based programmes requires an ecological perspective that looks at individual, interpersonal and environmental levels of the community [6].

Using an intervention mapping (IM) approach in planning health promotion programmes encompasses aspects of community participation in programme development and acknowledges the role of behavioural and environmental factors in health outcomes [7]. IM is grounded on a systematic approach in the development of health promotion programmes and the use of theories guiding strategies for change that are linked to behavioural and environmental factors related to health outcomes [7–9]. The approach is comprehensive, comprising six steps (1) a needs assessment, (2) design of a matrix of proximal programme objectives, (3) selection of theory-based intervention methods and practical applications (4) design of the programme, (5) adoption and implementation plans, and (6) programme evaluation plans [10, 11]. A number of public health programmes using an IM approach have been previously documented [7, 9, 12–18], however, less is known in regards to the use of IM in community-tailored interventions for malaria control and/or elimination. This paper documents the development, implementation and evaluation of a community-based malaria elimination project using an IM approach.

## Methods

### Project settings

The project has been implemented since 2012 in Ruhuha sector of the Bugesera district, Eastern province of Rwanda, located 42 km from Kigali, the capital city of Rwanda. The sector covers 54 km^2^ with a population estimated at 23,893 individuals living in 5098 households. Administratively, the sector is made up of five administrative cells, divided into 35 villages. The area is a moderate malaria endemic zone (two peaks occur year round) mainly due to its geographical characteristics such as altitude, marshes and swamps, and rice field irrigation schemes and cross border movements [3].

### Malaria elimination programme for Ruhuha (MEPR)

The MEPR was formed with the primary goal to contribute to the knowledge base for malaria elimination using community participatory approaches while building capacity of local scientists in the field of behavioural sciences, biomedical sciences, medical entomology and health economics. Pre-existing collaboration in malaria control studies between project members and local partners guided the selection of the site to deploy interventions targeting elimination.

#### Step 1: needs assessment: psychosocial determinants of preventive behaviours and environmental determinants of malaria

A formative analysis that includes open space discussions, a stakeholder analysis and household and entomological surveys were done to inform systematic development of targeted project interventions for behavioural and environmental changes [19–21]. Open space discussions highlighted actions to be emphasized in malaria elimination. A stakeholder analysis identified potential MEPR collaborators from various existing actors in the field of malaria control [22]. Household and entomological surveys included the analysis of psychosocial antecedents (also called determinants) of behaviour (attitudes, knowledge and practices), and biomedical, economic, environmental, and entomological aspects of malaria at household level. Acceptability and use of LLINs and IRS, ownership of community based health insurance (CBHI) to increase use of health services, malaria prevalence, mosquito density and behaviours were assessed, among others.

### Open space technology

Open space is a technique designed to help the members of a defined community reflect on an issue at hand, identify opportunities for change and set priorities among action steps to achieve desired goals in an innovative and productive way [23]. The approach is based on voluntary participation and participants should be interested in the topic of discussions with a certain degree of freedom and responsibility to facilitate exploration and experimentation as part of the process [23]. Two sessions were organized in 2010 and 2011 respectively as part of the MEPR needs assessment; details are provided elsewhere [19]. The first session explored different ways in which the community could contribute towards malaria reduction while the second looked at how community could contribute towards malaria elimination. During the open space process, MEPR team was not only gathering the information from the community but also participated in the discussions. As per the rule of the open space, agenda was set by the participants in relation to the topic to be discussed. Based on emerging subtopics, groups were formed and participants signed up for the groups with which they wished to be involved and were allowed to move from one group to another once they felt no more contribution or learning was achieved by continued participation in the same group [19].

### Stakeholder analysis

Malaria elimination project team in collaboration with local administrative (at sector level) and health authorities (national malaria control programme and local health centre) generated an initial list of other stakeholders in addition to participants attending open space discussions. The aim was to identify relevant stakeholders in malaria prevention and control and their potential roles/contributions towards MEPR’s goal. Using a snowball technique, further stakeholders were added and individual and group interviews were conducted. Stakeholders were asked about their malaria prevention and treatment activities, awareness of the MEPR, and their suggestions for participatory actions [22].

### Integrated household survey

A baseline household survey followed by an annual follow up survey was conducted to provide a thorough understanding of the needs of the community and malaria preventive and risk behaviours as well as the underlying environmental and psychosocial determinants. Secondly, the surveys explored demographic data, household health care-seeking practices and use of health services. Blood samples were collected to determine malaria epidemiology while mosquito density and identification of species were regularly analysed through entomological studies done using CDC light traps for adult mosquito and ‘dipping’ for mosquito larvae [24, 25].

#### Step 2: matrix of change objectives

Based on the needs assessment findings, which highlighted stakeholders to engage, behavioural and environmental malaria related gaps and actions to be taken, the second step of IM characterized behavioural and environmental outcomes as well as their determinants. Performance objectives defined as expected actions of the intervention were therefore operationalized for each determinant of the desired outcome [11]. Barriers associated with performing enumerated behaviours to achieve desired outcomes were highlighted and transformed into specific change objectives [16]. Specifically, outcomes of the needs assessment were discussed in community meetings attended by 110 community members (local administrative and health authorities, CHWs and youth representatives). Sessions included a “gallery walk” to allow participants move around and gain more knowledge in terms of specific findings per each programme component guided by each of the four MEPR PhDs (behavioural, biomedical, entomological, and health economics). Group discussions were subsequently organized during the meetings to inform decisions on desired project outcomes for behaviour change.

#### Step 3: selecting theoretical methods, practical applications and parameters for use

The third step in programme development involved the choice of theoretical methods, their practical applications and parameters for use needed to influence the determinants of behaviours and environmental conditions, based on empirical and context evidence [11, 26]. Theoretical methods are defined as general constructs derived from empirical research or theories and are used to explain and realize programme outcomes (i.e. consciousness raising). Practical applications refer to specific operationalization of the methods into practical use (i.e. findings dissemination meetings) while parameters for use are defined as required characteristics for a practical application to reflect a theoretical method [11, 26].

#### Step 4: design of programme components and materials

In collaboration with stakeholders identified in step one, the fourth step involved the development of programme materials, scope, sequence of activities and delivery channels in relation to theoretical methods and practical applications elaborated in step three as well as change objectives mentioned in step two. Lastly, the pretesting of elaborated programme materials was performed before final production.

#### Step 5: adoption, implementation and maintenance of innovative programmes

The fifth IM step involved an adoption and implementation plan of programme components as well as potential strategies for maintenance/institutionalization within routine local malaria control activities [11]. Based on the stakeholder analysis exercise conducted in step one, the fifth step identified relevant stakeholders to participate in the adoption and implementation of the innovative interventions. In addition, intervention outcomes and performance objectives for adoption, implementation and maintenance were enumerated [11].

#### Step 6: programme monitoring and evaluation plan

It is important to monitor the implementation and outcomes of the programme for the purposes of generating feedback for programme improvement and generating lessons for replication [27]. The last step of IM involved the development of evaluation objectives and the selection of process and outcome indicators in relation to the performance and change objectives highlighted in step two and theoretical methods and practical applications highlighted in step three [16]. Key process evaluation components proposed by Linnan and Steckler [27] were taken into consideration for both project components; the reach (exposure) and intensity of programme components delivered, the extent to which the programme was delivered as intended, the extent to which the programme was implemented and received, and approaches used to attract programme participants. The aim of the evaluation was mainly to estimate possible changes in regard to behavioural and environmental outcomes based on change objectives. Consequently, a follow up household survey was conducted in December 2014 among a random sample of 1410 households [28, 29], complemented by an end line qualitative study that was conducted in October 2015 and attended by 92 community representatives. Both studies explored current community perceptions of malaria burden, the level of CBHI ownership, changes in psychosocial factors including knowledge, attitudes and practices towards LLINs and IRS, process and outcome indicators in regards to the behavioural change intervention while the larval source control intervention was only evaluated during the final qualitative study.

## Results

### Step 1: needs assessment

In total, two open space meetings were held with community representatives; 62 and 82 participants in 2012 and 2013, respectively. Participants perceived malaria as a health concern and expressed willingness to contribute towards its elimination. Active demand at household and community level for diagnosis and prompt malaria case management were highlighted as key to reducing malaria when deployed in addition to the use of preventive measures such as LLINs and IRS. Malaria clubs were suggested as platforms to provide malaria messages to the wider community [19].

A stakeholder analysis that was conducted in 2013 using mixed methods generated a list of potential malaria actors who were further categorized into primary (lay community), secondary (local administrative and health institutions) and key stakeholders (donors and policy makers). Discussions suggested potential areas of contribution including promotion of malaria preventive measures and participation in the MEPR planning, implementation and knowledge translation activities [22].

The baseline household survey conducted in 2013 reported 92.8 and 94.5% coverage of LLINs and IRS, respectively [21]. However, bedbug infestation of LLINs and perceptions that IRS activated mosquitos, rather than killing them were enumerated as hindrances for use in a series of focus group discussions [20]. Furthermore, only two-thirds of the population was found to have a community based health insurance (CBHI) at baseline while malariometric data showed a 5% malaria parasite carriage prevalence (asymptomatic) and 13% of households had at least one member with malaria parasitemia [20, 21].

An entomological monitoring survey reported 46.4 and 3.3% *Anopheles gambiae* s.l. (main malaria vector collected) of the total mosquitoes collected in 2013 and 2014, respectively. However, a general increase in mosquito density was observed as opposed to a decrease of *Anopheles gambiae* density in 2013 and 2014 and an average of 18 and 35.3 mosquitoes per house surveyed was reported, respectively.

### Step 2: synthesis of determinants and defining change objectives

The envisioned overall project outcome was to contribute towards achieving malaria elimination in Ruhuha sector within the project lifespan in collaboration with the community at large. Changeable behavioural determinants were enumerated and subsequently behavioural outcomes included promotion of malaria preventive measures such as (1) to increase the correct and consistent use of LLINs, (2) to accept IRS at household level including master and storage rooms, (3) to clear peridomestic mosquito breeding sites and (4) to promote prompt care-seeking for fever cases by increasing awareness and recognition of malaria symptoms and CBHI ownership. Changeable environmental outcomes included the larval source control in local rice paddies, marshlands and peridomestic water dams. For each highlighted outcome, a number of performance objectives stating what should be done were accordingly identified as outlined in Table 1.

**Table 1:** Programme outcomes and performance objectives

Behavioural and environmental outcomes	Performance objectives
Increased correct and consistent use of LLINs	To own LLINs
	To use LLINs every night
	Targeted actions to prevent/eliminate bedbugs infestation
IRS acceptance at household level	To allow IRS at household level
	To allow IRS in master room
	To allow IRS in storage rooms
Prevention of peridomestic mosquito breeding	To cover households water collection instruments
	To clear breeding sites surrounding homesteads
Prompt care-seeking	To have a health insurance
	To learn and identify malaria symptoms to enable early recognition of malaria fever
	To practice early care-seeking (within 24 h)
	To create awareness on the importance of diagnostic testing before treatment
	To create awareness on the importance of treatment adherence (taking and completing malaria medication as instructed by providers)
Mosquito larval source control in local rice paddies/marshlands	To select the implementation team
	To ensure proper training before implementation
	To adhere to an implementation plan

The last step included the creation of a matrix of change objectives based on performance objectives and the determinants (Table 2).

**Table 2:** Addressing determinants of performance objectives

Performance objectives	Determinants	
	Information/knowledge	Self-efficacy/skills
To own LLINs	Explain the benefits of LLINs ownership	Demonstrate how a net should be used
To fight bedbugs	Explain the importance of fighting bedbugs as hindrances of LLINs use	Demonstrate how the technique should be done using hot water and local laundry powder soap (OMO)
To use LLINs every night	Explain the importance of using LLINs every night	Use of LLINs in spite of warm weather or weather changes
To allow IRS at household level	Describe IRS benefits	Allow IRS in master room Allow IRS in storage rooms
To cover households water collection instruments	Explain how water collection instruments can be a source of mosquito breeding.	Cover water collection instruments every time
To clear mosquito breeding sites surrounding homesteads	Explain the importance of clearing water pits and bushes surrounding the homesteads	Perform regular cleaning activities at household level
To own a health insurance	Describe the importance of a health insurance in prompt health care-seeking	Participate in solidarity groups to enable regular contribution
To provide education on malaria symptoms	Describe common malaria symptoms	Identify common malaria symptoms
To promote early fever/malaria care-seeking (within 24 h)	Describe the importance of early care-seeking	Take a decision of seeking care promptly
To raise community awareness on the importance of diagnostic test before treatment	Describe the importance of a lab confirmed treatment for proper care and prevention of treatment resistance	Seek care at health facility (health centre/hospital)
To raise community awareness on the importance of malaria medication compliance	Describe the importance of treatment compliance to prevent disease relapse and treatment resistance	Adhere to the advice/treatment provided
To ensure proper training before implementation of larval source control	To provide knowledge on mosquito reproduction, malaria preventive measures and larval source control using biological substances	Attend onsite practical training
To adhere to the implementation plan	Discuss how larval source control will be implemented To explain the use of reporting formats	Participate in actual implementation of larval source control

### Step 3: theoretical methods, practical applications and parameters for use

In IM step 3, theoretical concepts and practical applications that guided programme implementation were defined and attributed to the parameters for use deemed changeable to produce positive effect on behaviours (Table 3).

**Table 3:** Programme methods, applications and parameters for use

Methods	Definition	Applications	Parameters for use
Theory of planned behaviour	Information to what extent attitudes, subjective norms and perceived behavioural control affect human behaviours	Annual surveys	Awareness, beliefs, social support, self-efficacy
Feedback (theories of learning)	Information to what extent people are performing and how performance is having impact	Annual dissemination meetings Weekly community meetings Monthly progress reports	Awareness, skills
Self-regulation	Controlling oneself through monitoring, goal setting, feedback instruction and social support	Open space	Awareness, SKILLS
Participation (diffusion of innovation theory)	Engagement of participants in problem solving, decision making and change activities	Needs assessment Planning Training Implementation Evaluation	Skills

### Step 4: design of programme components and materials

Based on the findings of the needs assessment, two programme components were deemed important. Firstly, a need to focus on behavioural aspects through community mobilization was identified, hence the establishment of community malaria action teams (CMATs) since mid-2014. CMATs were established to mobilize the local community on malaria preventive measures. Secondly, trained community members (mainly rice farmers’ cooperatives and CMATs) deployed an environmental intervention using biological substances “*Bacillus thuringiensis israelensis* (Bti)” for mosquito larval control across mapped and newly identified breeding sites from February to July 2015.

#### Community malaria action teams (CMATs)

The Ruhuha health centre, the Ruhuha administrative sector and MEPR collaboratively proposed inclusion criteria for members of CMATs platforms. Ruhuha sector has 35 villages and three members at village level were included; a village leader, a community health work and a youth representative. A village leader was selected as one of the most influential people, a community health worker was included as the person in charge of community health and a youth representative was included to mobilize a large group of the community (youth).

In a results dissemination workshop conducted in May 2014, CMATs members discussed and agreed on their terms of reference which included identification of local malaria related issues, organization of community outreach activities in response to the issues identified, provision of feedback to both communities and research team, monthly progress reporting of the programme activities, and the evaluation of implemented programmes. Expected behavioural and environmental outcomes were listed, including increased correct and consistent use of IVM strategies such LLINs and IRS, clearing of mosquito breeding sites and promotion of prompt health care-seeking. The MEPR team translated the outcomes of CMATs discussions and agreed action points into a small brochure that was shared with CMATs for validation and later on distributed for daily use. To increase knowledge and skills among CMATs members, the MEPR team organized trainings on various topics about malaria transmission, prevention and treatment.

CMATs conducted monthly educational sessions through interpersonal community meetings and/or one-to-one home visits. Though CMATs used pre-identified themes (brochures) during their educational sessions, contextual factors were taken into consideration to guide the focus of discussions (i.e. existence of mosquito breeding sites around homesteads). Monthly progress reports submitted to the sector level included description of planned activities, performed activities, geographical zone covered and challenges faced during implementation.

#### Mosquito larval source control intervention

The mosquito larval source control intervention was informed by a prior mixed methods socio-economic survey conducted in January 2015 to explore community awareness, acceptance and willingness to invest in larviciding. The socio-economic survey highlighted a need to engage local beneficiaries in the planning, implementation and evaluation phases. Although a majority of participants in the socio-economic survey were unaware of such an innovation as part of malaria control strategies, they deemed the intervention important to combine with existing malaria preventive strategies. Only few participants expressed concerns with regard to safety and effectiveness of the intervention. Willingness to invest in the intervention—if proven effective—was more in a sense of labor time while proposed monetary contributions would cover only one-fourth of the full intervention cost, hence the need to study other financing mechanisms.

MEPR closely collaborated with local health and administrative authorities, local rice farmers’ cooperatives and CMATs members during planning and implementation of the intervention. A pre-intervention training was organized and conducted for the spraying and mosquito monitoring teams. The actual intervention implementation included 18 weekly application (spraying) rounds of biological larvicide “Bti” for mosquito larval control in local marshlands mainly occupied by rice fields and peridomestic water dams of Ruhuha sector. Larvae and adult mosquitoes were regularly monitored for evaluation purposes.

### Step 5: programme adoption, implementation and maintenance

In addition to identification of stakeholders to be engaged, step five of IM in the project outlined activities to be performed for adoption, implementation and maintenance of the project components: community education through CMATs and the mosquito larval source control (Table 4).

**Table 4:** Specific activities for programme adoption, implementation and maintenance

	Adoption	Implementation	Maintenance
Performance objectives for community mobilization through CMATs	Local administrative and health authorities to be informed on and allow the establishment of CMATs platforms	Selection and training of CMATs Malaria preventive messages through group/one-to-one meetings by CMATs Monthly reports by CMATs	Malaria preventive messages through group/one-to-one meetings by CMATs Health centre regular support visits and ongoing trainings to CMATs CMATs monthly reports to the health centre
Performance objectives for the mosquito larval source control intervention	Local administrative, health authorities, CMATs and rice farmers cooperatives to be informed on and allow the implementation of Bti intervention	Training and implementation of Bti intervention by rice farmers and CMATs	Rice farmers’ cooperatives to contribute to the funding of Bti intervention in future Local community to provide labor time for future LSM using Bti National malaria control programmes to adopt the intervention into integrated vector management strategies

### Step 6: process and outcome evaluation

Based on the composition of their members, the project CMATs were seen as crucial for malaria prevention by the local community. Six months following their establishment, a follow up household survey conducted in December 2014 reported that 39% of community members were visited by at least one CMATs member while 52 and 38% reported receiving messages on clearing peridomestic breeding sites and proper use of LLINs, respectively. At least 23, 18 and 13% of the respondents reported receiving messages on early health care-seeking, importance of a CBHI and the benefits of IRS, respectively. However, participants highlighted that messages were mainly provided by CHWs members of CMATs in collaboration with village leaders while youth involvement was perceived as minimal due to frequent absence in planned malaria related activities.

On one hand, while CMATs reports were found resourceful and consistent in the beginning, some delays and inconsistencies were observed by the MEPR team and discussed in planned quarterly meetings held between CMATs and MEPR. It was therefore agreed that progress reports should be brought to the local health centre instead of to the local administrative sector, which improved both regularity and consistency of reports. On the other hand, CMATs also reported challenges related to the implementation of their activities including low personal malaria risk perception among community members despite acknowledgement that malaria is a problem, low level of community attendance in educational sessions, insufficient didactical materials, long travelling distance and sometimes absence of household members during the mainly impromptu home visits, among others.

A household follow up survey in 2014 reported an increase from 94.5 to 98.7% in the coverage of IRS while LLINs coverage remained without change. A high increase from 61.3 to 91% in CBHI membership when compared to the 2013 baseline survey was also reported. Additionally, prompt and adequate care among households with presumed malaria cases (estimated at 21% compared to 68% reported in 2013) was determined by high self-efficacy, recognition of malaria symptoms and ownership of a CBHI [22, 28].

An end line qualitative study conducted in October 2015 highlighted a general perception of reduction of malaria during MEPR. The reduction was attributed to the improved sensitization towards malaria control measures that contributed to an increase in community knowledge, acceptance and use of vector control strategies and increase in the coverage of CBHI. The same study reported an increased positive awareness with regard to the implemented LSM intervention as opposed to baseline hence a reduction in mosquito nuisance and bites was commonly reported by those actively engaged and the community at large.

Furthermore, entomological monitoring and evaluation surveys conducted every two weeks during the larval source control programme implementation supported community perceptions and a reduction in adult mosquito density generally; and late stage (3 and 4) and pupa of *Anopheles* sp. specifically in treated sites when compared to control site was noted. Challenges related to geographical and operational factors were however enumerated during the end line study including heavy rains and deep and slippery marshlands that are hard to cover by manual application and inadequate staff during implementation, hence participants suggested that challenges should be taken into cognizance prior to future scale up. In addition, the sustainability of the intervention was highlighted as a challenge, thus suggestions for stakeholder participation in searching for strategies that involve both communities and government were made by participants to ensure continuation of larval source management. In spite of intervention benefits reported, participants also highlighted a clear need for ongoing sensitization on existing malaria control measures to prevent an eventual relapse.

## Discussion

The current paper described the development process, content and evaluation of the malaria elimination project using an intervention mapping approach. MEPR used an interactive and ecological approach whereby community and researchers exchanged information through consultative assessment of needs and agreed actionable recommendations were collaboratively translated into targeted implementation plans. Behavioural and environmental determinants and outcome measurements were hence identified to tackle malaria related aspects including the promotion of malaria preventive measures and a larval source control intervention.

MEPR progressed from identification of stakeholders as a prerequisite to ensure wide-ranging representation of various perspectives as also suggested elsewhere [30, 31]. Formative research in the area highlighted malaria as a health concern with various factors contributing to its transmission including poverty, perceived ineffectiveness and misuse of existing malaria preventive measures as well as environmental factors such as the presence of a number of marshlands where malaria vectors are abundant.

As similarly indicated elsewhere, poverty is having a pervasive effect in exposing people to infection by living in houses that are prone to mosquito proliferation in addition to medical services costs that are prohibitive to poor households and hinder prompt care-seeking, hence increase severe and fatal malaria complications [32]. However, the ownership of a CBHI has been highlighted as a promoting factor for early health care-seeking, closing the gap between low and high income population in relation to the access of health care services; hence mechanisms to optimize its coverage should be explored [33, 34].

The alternative and/or inconsistent LLIN use resulting from perceived physical discomfort and perceived limited efficacy of IRS against malaria vectors have been observed previously as hampering malaria control efforts, and hence suggesting a behaviour change communication campaign that ensures acceptable participatory mechanisms for appropriate use [35–38].

The creation of local CMATs platforms intensified community sensitization for the use and acceptance of IVM strategies, prompt care-seeking and clean environment which is consistent with previous studies that demonstrated the instrumental role of community educational groups in raising correct knowledge about malaria control and the use of health services [39–41]. Active community participation through these platforms in prioritizing malaria related messages to be developed and activities to be deployed was then found rewarding, created a conducive environment for ownership and utilization of knowledge produced at individual and village levels in relation to health [40, 42–44].

As similarly shown in Ghana, the use of CMATs was however not without challenges, specifically in relation to difficulties in combining both volunteer and everyday work [45]. In addition, CMATs have used a community-oriented approach in which self-organization was a key element. However, the roll back malaria suggests that community-based activities need to be supervised, guided and assured of quality to ensure effectiveness [46]. It is therefore recommended that community related projects design strategies that inclusively ensure community initiatives are widely deployed by community members but also receive regular training and support visits from the local health authorities.

The implementation of the larval source control intervention in local marshlands and other peridomestic breeding sites using biological substances (Bti) led to a reduction in mosquito density and nuisance biting while contributing to the local empowerment, ownership and increased local knowledge in larval source control. Involving community members such as rice farmers’ cooperatives, who are part of the social system throughout the intervention resulted into clearly observed effects [44, 47]. The use larval source control to improve outcomes in malaria control is in accordance to what has been mainly reported in Tanzania [43]. Furthermore, the WHO highlights the importance of identifying strategies that are acceptable to the people they affect, and that can be integrated into their daily lives and community structures to achieve effective community participation [48]. However, maintaining the hard-won gains is perceived as challenging especially when continuation of activities does not solely depend on community participation alone, but also involve multi-sectoral collaboration.

The fact that the larval source control programme reduces mosquito density and nuisance may lead to low malaria risk perception in the community, hence negative implications on existing IVM strategies such as LLINs use and IRS acceptability. For effective and sustainable malaria prevention strategies, the vital role of community participation and perception about the benefits of comprehensive malaria control as well as addressing community emerging attitudes on innovative malaria control tools should therefore be emphasized [49]. Health education targeting such community perceptions and behaviours has been previously proven as a key element in community-based malaria control interventions for elimination [36, 50, 51].

The use of IM approach in MEPR guided the process of interventions development based on theory and local context to respond to community needs [17, 52]. IM also guided the ecological perspective adopted by MEPR—encompassing both personal and environmental determinants of malaria during project interventions [9]. This has enabled the project to design and implement interventions in a way that increases the likelihood of acceptance and maximizes the benefit to the community [53]. IM also enabled documentation of intervention content from initial phase to inform future replication and/or improvement as also suggested elsewhere [54].

MEPR demonstrated that IM is a feasible and helpful method for providing an evidence based, and theoretical structure to complex malaria behavioural and environmental change interventions [16]. While thinking of using IM in future studies however, authors may consider possessing basic knowledge of behavioural theories and be aware of the IM systematic approach that includes technical aspects when compared to the usual public health interventions as also demonstrated elsewhere [14, 16, 52].

While MEPR baseline studies included an active identification of malaria cases, due to time and financial constraints, the project was however unable to conduct a final analysis to serve as impact evaluation thus making comparison of malaria trends critical. This part of the project is, as equally important and future similar programmes need to ensure that malaria clinical evaluation is part of the programme design to validate behavioural and environmental changes throughout the programme. Nevertheless, the local health centre routine data indicated an 11.5% of malaria burden in 2015 as compared to 27% reported in 2012.

Rwanda has a strongly organized CHWs system that plays a significant role in malaria prevention and case management [55]. This implies that highlighted achievements in community mobilization during project lifespan should not be solely attributed to the MEPR efforts. However, as health related programmes are not regarded as the responsibility of health workers alone, preventive interventions as initiated by the MEPR project in which community members such as village leaders, youth, rice farmers and other community stakeholders play a key role, should be further improved, implemented on a broader scale and systematically evaluated.

## Conclusion

MEPR is the first to deploy an intervention-mapping framework with two key-components targeting behavioural and environmental factors conducive to malaria transmission. The framework was beneficial in a sense that it allowed a systematic learning and adaptation of interventions from planning to evaluation phases. The programme hence contributed to reported increase in community acceptance and use of vector control strategies and coverage of CBHI. The deployment of the larval source control intervention through a participatory process that entails close consultation, collaborative monitoring, decision-making and flexibility also contributed to the reduction of mosquito abundance and nuisance biting while contributing to community empowerment with knowledge and skills. Consistent monitoring, learning, adaptation and commitment are therefore key elements to sustain gains observed and inform effective interventions as malaria dynamics change.

## Abbreviations

ACTs: artemisinin based combination therapy
BTI: *Bacillus thuringiensis Israelensis*
CBHI: community-based health insurance
CHWs: community health workers
CMATs: community malaria action teams
IM: intervention mapping
IRS: indoor residual spraying
IVM: integrated vector management
LLIN: long lasting insecticide treated nets
MEPR: malaria elimination programme for Ruhuha
WHO: World Health Organization
